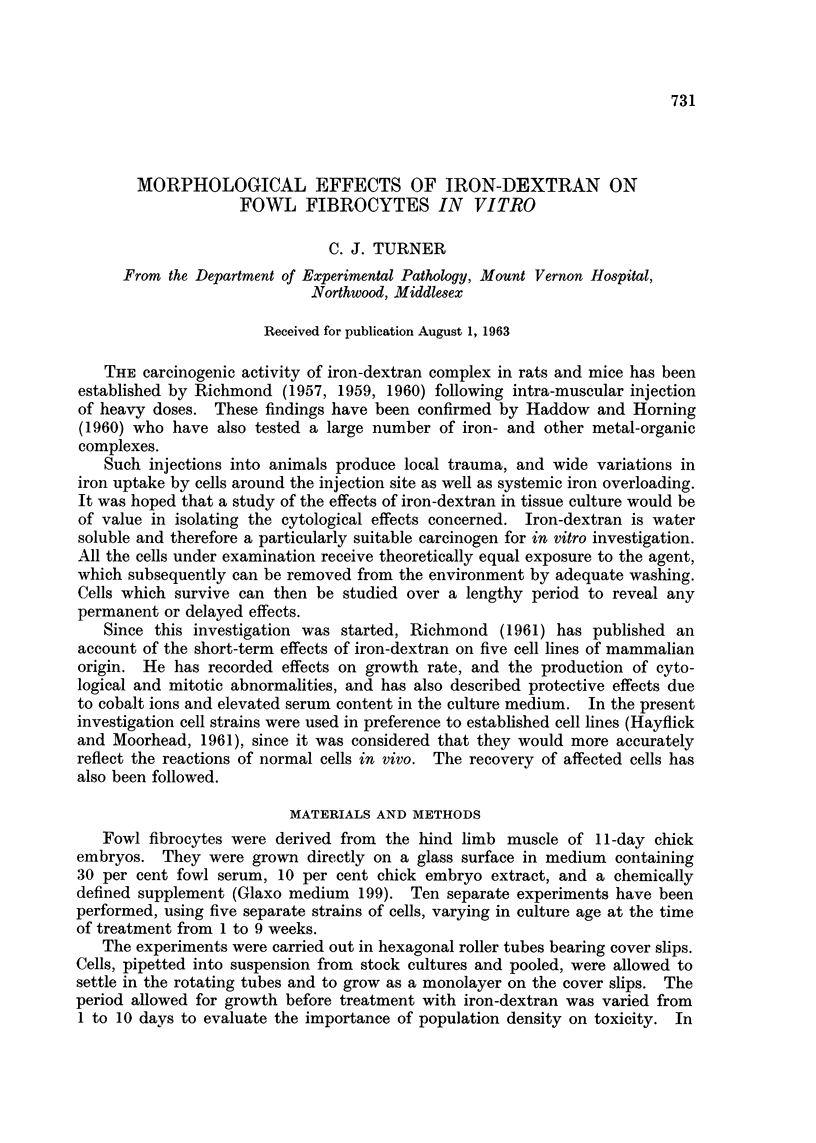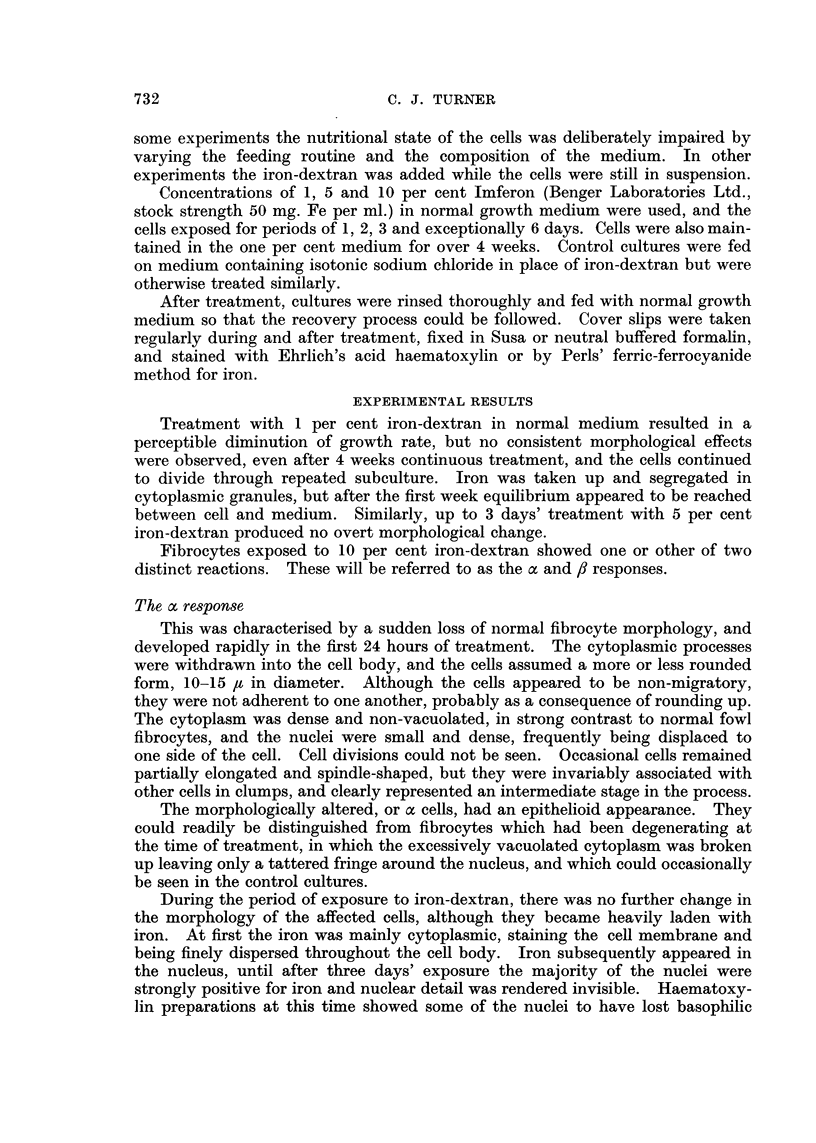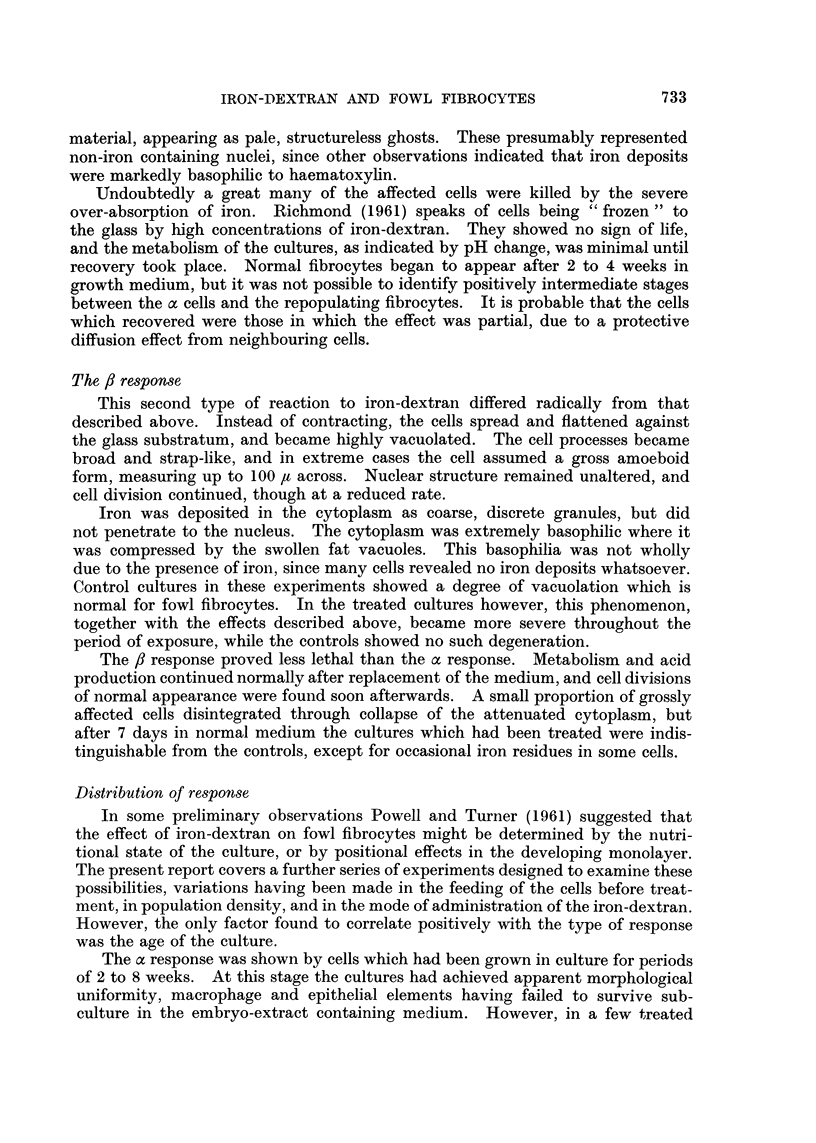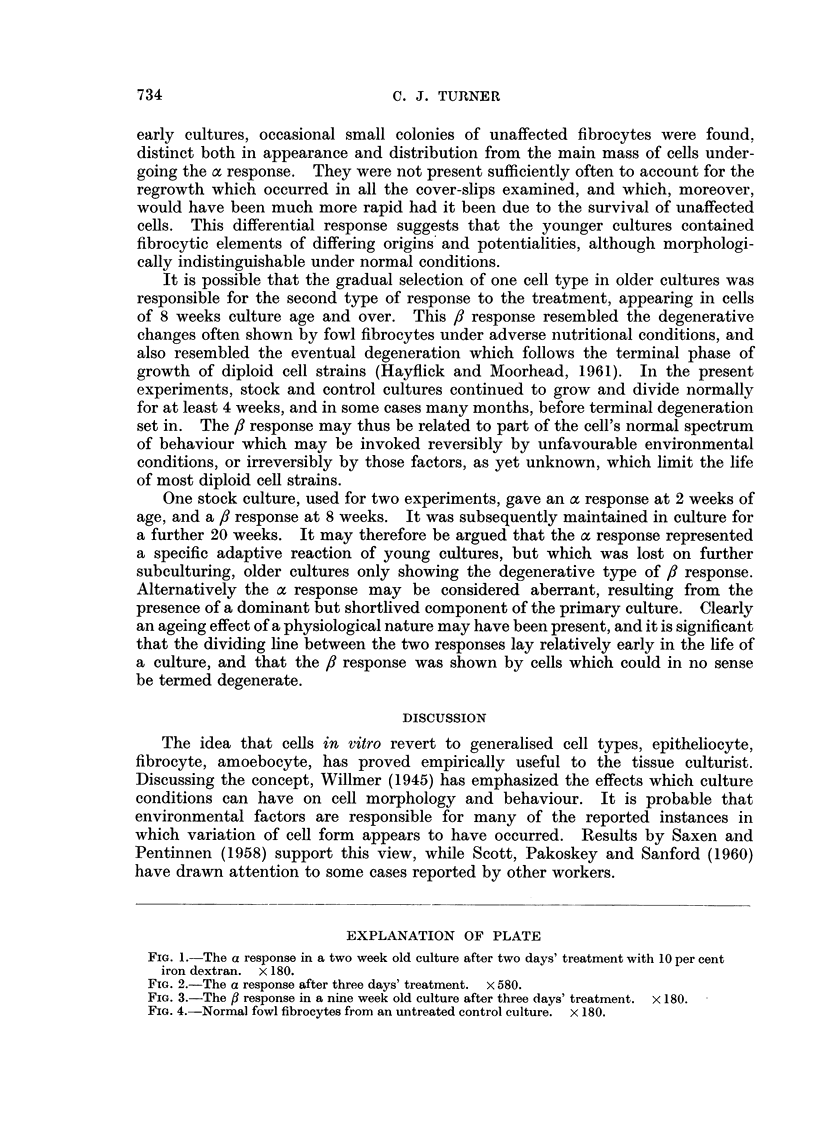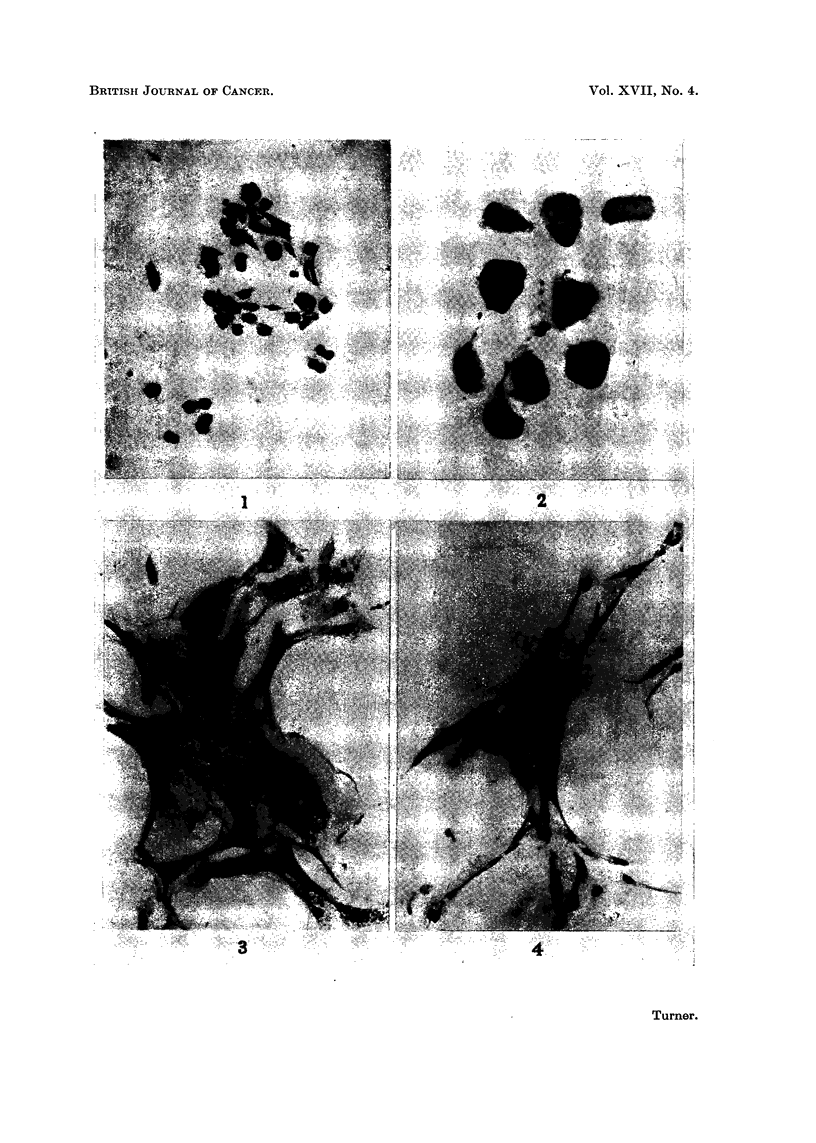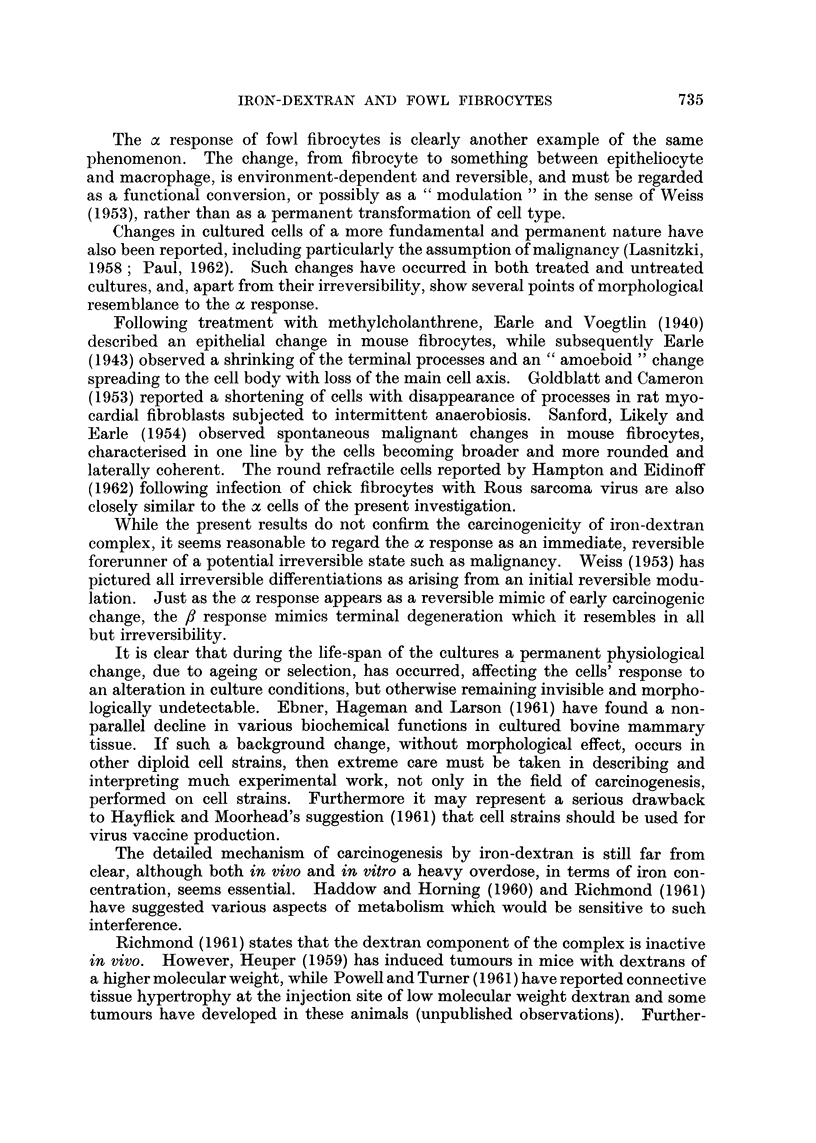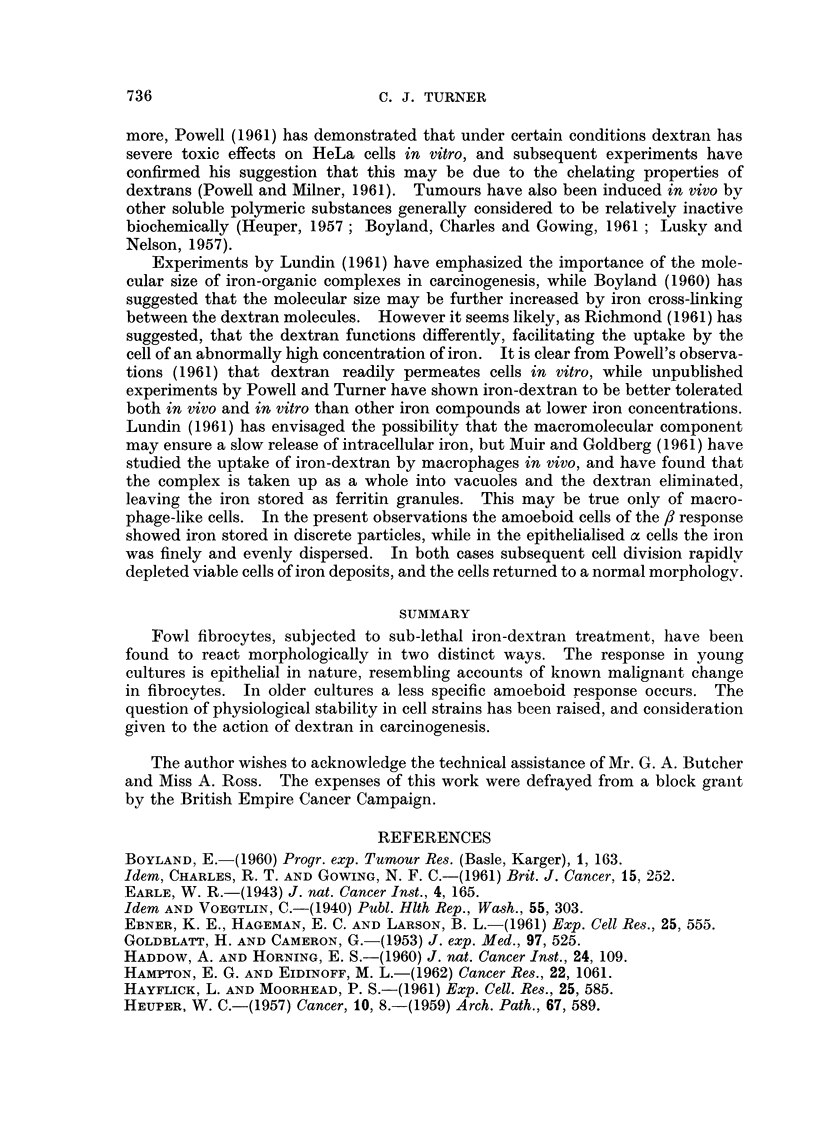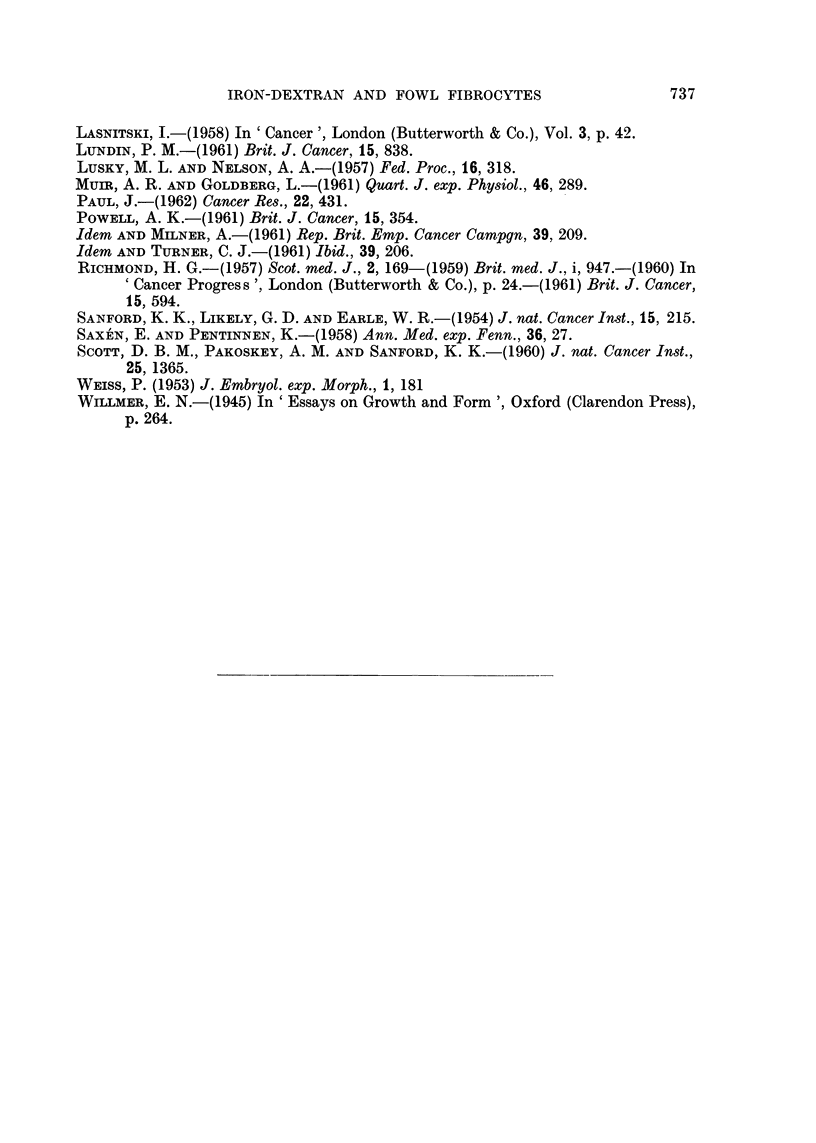# Morphological Effects of Iron-Dextran on Fowl Fibrocytes in Vitro

**DOI:** 10.1038/bjc.1963.94

**Published:** 1963-12

**Authors:** C. J. Turner

## Abstract

**Images:**


### MORPHOLOGICAL EFFECTS OF IRON-DEXTRAN ON FOWL FIBROCYTES *IN VITRO*

C. J. TURNER

*From the Department of Experimental Pathology, Mount Vernon Hospital, Northwood, Middlesex*

Received for publication August 1, 1963

THE carcinogenic activity of iron-dextran complex in rats and mice has been established by Richmond (1957, 1959, 1960) following intra-muscular injection of heavy doses. These findings have been confirmed by Haddow and Horning (1960) who have also tested a large number of iron- and other metal-organic complexes.

Such injections into animals produce local trauma, and wide variations in iron uptake by cells around the injection site as well as systemic iron overloading. It was hoped that a study of the effects of iron-dextran in tissue culture would be of value in isolating the cytological effects concerned. Iron-dextran is water soluble and therefore a particularly suitable carcinogen for *in vitro* investigation. All the cells under examination receive theoretically equal exposure to the agent, which subsequently can be removed from the environment by adequate washing. Cells which survive can then be studied over a lengthy period to reveal any permanent or delayed effects.

Since this investigation was started, Richmond (1961) has published an account of the short-term effects of iron-dextran on five cell lines of mammalian origin. He has recorded effects on growth rate, and the production of cytological and mitotic abnormalities, and has also described protective effects due to cobalt ions and elevated serum content in the culture medium. In the present investigation cell strains were used in preference to established cell lines (Hayflick and Moorhead, 1961), since it was considered that they would more accurately reflect the reactions of normal cells *in vivo*. The recovery of affected cells has also been followed.

### MATERIALS AND METHODS

Fowl fibrocytes were derived from the hind limb muscle of 11-day chick embryos. They were grown directly on a glass surface in medium containing 30 per cent fowl serum, 10 per cent chick embryo extract, and a chemically defined supplement (Glaxo medium 199). Ten separate experiments have been performed, using five separate strains of cells, varying in culture age at the time of treatment from 1 to 9 weeks.

The experiments were carried out in hexagonal roller tubes bearing cover slips. Cells, pipetted into suspension from stock cultures and pooled, were allowed to settle in the rotating tubes and to grow as a monolayer on the cover slips. The period allowed for growth before treatment with iron-dextran was varied from 1 to 10 days to evaluate the importance of population density on toxicity. In some experiments the nutritional state of the cells was deliberately impaired by varying the feeding routine and the composition of the medium. In other experiments the iron-dextran was added while the cells were still in suspension.

Concentrations of 1, 5 and 10 per cent Imferon (Benger Laboratories Ltd., stock strength 50 mg. Fe per ml.) in normal growth medium were used, and the cells exposed for periods of 1, 2, 3 and exceptionally 6 days. Cells were also maintained in the one per cent medium for over 4 weeks. Control cultures were fed on medium containing isotonic sodium chloride in place of iron-dextran but were otherwise treated similarly.

After treatment, cultures were rinsed thoroughly and fed with normal growth medium so that the recovery process could be followed. Cover slips were taken regularly during and after treatment, fixed in Susa or neutral buffered formalin, and stained with Ehrlich's acid haematoxylin or by Perls' ferric-ferrocyanide method for iron.

### EXPERIMENTAL RESULTS

Treatment with 1 per cent iron-dextran in normal medium resulted in a perceptible diminution of growth rate, but no consistent morphological effects were observed, even after 4 weeks continuous treatment, and the cells continued to divide through repeated subculture. Iron was taken up and segregated in cytoplasmic granules, but after the first week equilibrium appeared to be reached between cell and medium. Similarly, up to 3 days' treatment with 5 per cent iron-dextran produced no overt morphological change.

Fibrocytes exposed to 10 per cent iron-dextran showed one or other of two distinct reactions. These will be referred to as the $\alpha$ and $\beta$ responses.

### *The $\alpha$ response*

This was characterised by a sudden loss of normal fibrocyte morphology, and developed rapidly in the first 24 hours of treatment. The cytoplasmic processes were withdrawn into the cell body, and the cells assumed a more or less rounded form, 10–15 $\mu$ in diameter. Although the cells appeared to be non-migratory, they were not adherent to one another, probably as a consequence of rounding up. The cytoplasm was dense and non-vacuolated, in strong contrast to normal fowl fibrocytes, and the nuclei were small and dense, frequently being displaced to one side of the cell. Cell divisions could not be seen. Occasional cells remained partially elongated and spindle-shaped, but they were invariably associated with other cells in clumps, and clearly represented an intermediate stage in the process.

The morphologically altered, or $\alpha$ cells, had an epithelioid appearance. They could readily be distinguished from fibrocytes which had been degenerating at the time of treatment, in which the excessively vacuolated cytoplasm was broken up leaving only a tattered fringe around the nucleus, and which could occasionally be seen in the control cultures.

During the period of exposure to iron-dextran, there was no further change in the morphology of the affected cells, although they became heavily laden with iron. At first the iron was mainly cytoplasmic, staining the cell membrane and being finely dispersed throughout the cell body. Iron subsequently appeared in the nucleus, until after three days' exposure the majority of the nuclei were strongly positive for iron and nuclear detail was rendered invisible. Haematoxylin preparations at this time showed some of the nuclei to have lost basophilic material, appearing as pale, structureless ghosts. These presumably represented non-iron containing nuclei, since other observations indicated that iron deposits were markedly basophilic to haematoxylin.

Undoubtedly a great many of the affected cells were killed by the severe over-absorption of iron. Richmond (1961) speaks of cells being " frozen " to the glass by high concentrations of iron-dextran. They showed no sign of life, and the metabolism of the cultures, as indicated by pH change, was minimal until recovery took place. Normal fibrocytes began to appear after 2 to 4 weeks in growth medium, but it was not possible to identify positively intermediate stages between the $\alpha$ cells and the repopulating fibrocytes. It is probable that the cells which recovered were those in which the effect was partial, due to a protective diffusion effect from neighbouring cells.

*The $\beta$ response*

This second type of reaction to iron-dextran differed radically from that described above. Instead of contracting, the cells spread and flattened against the glass substratum, and became highly vacuolated. The cell processes became broad and strap-like, and in extreme cases the cell assumed a gross amoeboid form, measuring up to 100 $\mu$ across. Nuclear structure remained unaltered, and cell division continued, though at a reduced rate.

Iron was deposited in the cytoplasm as coarse, discrete granules, but did not penetrate to the nucleus. The cytoplasm was extremely basophilic where it was compressed by the swollen fat vacuoles. This basophilia was not wholly due to the presence of iron, since many cells revealed no iron deposits whatsoever. Control cultures in these experiments showed a degree of vacuolation which is normal for fowl fibrocytes. In the treated cultures however, this phenomenon, together with the effects described above, became more severe throughout the period of exposure, while the controls showed no such degeneration.

The $\beta$ response proved less lethal than the $\alpha$ response. Metabolism and acid production continued normally after replacement of the medium, and cell divisions of normal appearance were found soon afterwards. A small proportion of grossly affected cells disintegrated through collapse of the attenuated cytoplasm, but after 7 days in normal medium the cultures which had been treated were indistinguishable from the controls, except for occasional iron residues in some cells.

*Distribution of response*

In some preliminary observations Powell and Turner (1961) suggested that the effect of iron-dextran on fowl fibrocytes might be determined by the nutritional state of the culture, or by positional effects in the developing monolayer. The present report covers a further series of experiments designed to examine these possibilities, variations having been made in the feeding of the cells before treatment, in population density, and in the mode of administration of the iron-dextran. However, the only factor found to correlate positively with the type of response was the age of the culture.

The $\alpha$ response was shown by cells which had been grown in culture for periods of 2 to 8 weeks. At this stage the cultures had achieved apparent morphological uniformity, macrophage and epithelial elements having failed to survive subculture in the embryo-extract containing medium. However, in a few treated early cultures, occasional small colonies of unaffected fibrocytes were found, distinct both in appearance and distribution from the main mass of cells undergoing the $\alpha$ response. They were not present sufficiently often to account for the regrowth which occurred in all the cover-slips examined, and which, moreover, would have been much more rapid had it been due to the survival of unaffected cells. This differential response suggests that the younger cultures contained fibrocytic elements of differing origins and potentialities, although morphologically indistinguishable under normal conditions.

It is possible that the gradual selection of one cell type in older cultures was responsible for the second type of response to the treatment, appearing in cells of 8 weeks culture age and over. This $\beta$ response resembled the degenerative changes often shown by fowl fibrocytes under adverse nutritional conditions, and also resembled the eventual degeneration which follows the terminal phase of growth of diploid cell strains (Hayflick and Moorhead, 1961). In the present experiments, stock and control cultures continued to grow and divide normally for at least 4 weeks, and in some cases many months, before terminal degeneration set in. The $\beta$ response may thus be related to part of the cell's normal spectrum of behaviour which may be invoked reversibly by unfavourable environmental conditions, or irreversibly by those factors, as yet unknown, which limit the life of most diploid cell strains.

One stock culture, used for two experiments, gave an $\alpha$ response at 2 weeks of age, and a $\beta$ response at 8 weeks. It was subsequently maintained in culture for a further 20 weeks. It may therefore be argued that the $\alpha$ response represented a specific adaptive reaction of young cultures, but which was lost on further subculturing, older cultures only showing the degenerative type of $\beta$ response. Alternatively the $\alpha$ response may be considered aberrant, resulting from the presence of a dominant but shortlived component of the primary culture. Clearly an ageing effect of a physiological nature may have been present, and it is significant that the dividing line between the two responses lay relatively early in the life of a culture, and that the $\beta$ response was shown by cells which could in no sense be termed degenerate.

### DISCUSSION

The idea that cells *in vitro* revert to generalised cell types, epitheliocyte, fibrocyte, amoebocyte, has proved empirically useful to the tissue culturist. Discussing the concept, Willmer (1945) has emphasized the effects which culture conditions can have on cell morphology and behaviour. It is probable that environmental factors are responsible for many of the reported instances in which variation of cell form appears to have occurred. Results by Saxen and Pentinnen (1958) support this view, while Scott, Pakoskey and Sanford (1960) have drawn attention to some cases reported by other workers.

---

### EXPLANATION OF PLATE

FIG. 1.—The $\alpha$ response in a two week old culture after two days' treatment with 10 per cent iron dextran. $\times 180$.

FIG. 2.—The $\alpha$ response after three days' treatment. $\times 580$.

FIG. 3.—The $\beta$ response in a nine week old culture after three days' treatment. $\times 180$.

FIG. 4.—Normal fowl fibrocytes from an untreated control culture. $\times 180$.

1

2

3

4

The $\alpha$ response of fowl fibrocytes is clearly another example of the same phenomenon. The change, from fibrocyte to something between epitheliocyte and macrophage, is environment-dependent and reversible, and must be regarded as a functional conversion, or possibly as a " modulation " in the sense of Weiss (1953), rather than as a permanent transformation of cell type.

Changes in cultured cells of a more fundamental and permanent nature have also been reported, including particularly the assumption of malignancy (Lasnitzki, 1958 ; Paul, 1962). Such changes have occurred in both treated and untreated cultures, and, apart from their irreversibility, show several points of morphological resemblance to the $\alpha$ response.

Following treatment with methylcholanthrene, Earle and Voegtlin (1940) described an epithelial change in mouse fibrocytes, while subsequently Earle (1943) observed a shrinking of the terminal processes and an " amoeboid " change spreading to the cell body with loss of the main cell axis. Goldblatt and Cameron (1953) reported a shortening of cells with disappearance of processes in rat myocardial fibroblasts subjected to intermittent anaerobiosis. Sanford, Likely and Earle (1954) observed spontaneous malignant changes in mouse fibrocytes, characterised in one line by the cells becoming broader and more rounded and laterally coherent. The round refractile cells reported by Hampton and Eidinoff (1962) following infection of chick fibrocytes with Rous sarcoma virus are also closely similar to the $\alpha$ cells of the present investigation.

While the present results do not confirm the carcinogenicity of iron-dextran complex, it seems reasonable to regard the $\alpha$ response as an immediate, reversible forerunner of a potential irreversible state such as malignancy. Weiss (1953) has pictured all irreversible differentiations as arising from an initial reversible modulation. Just as the $\alpha$ response appears as a reversible mimic of early carcinogenic change, the $\beta$ response mimics terminal degeneration which it resembles in all but irreversibility.

It is clear that during the life-span of the cultures a permanent physiological change, due to ageing or selection, has occurred, affecting the cells' response to an alteration in culture conditions, but otherwise remaining invisible and morphologically undetectable. Ebner, Hageman and Larson (1961) have found a non-parallel decline in various biochemical functions in cultured bovine mammary tissue. If such a background change, without morphological effect, occurs in other diploid cell strains, then extreme care must be taken in describing and interpreting much experimental work, not only in the field of carcinogenesis, performed on cell strains. Furthermore it may represent a serious drawback to Hayflick and Moorhead's suggestion (1961) that cell strains should be used for virus vaccine production.

The detailed mechanism of carcinogenesis by iron-dextran is still far from clear, although both *in vivo* and *in vitro* a heavy overdose, in terms of iron concentration, seems essential. Haddow and Horning (1960) and Richmond (1961) have suggested various aspects of metabolism which would be sensitive to such interference.

Richmond (1961) states that the dextran component of the complex is inactive *in vivo*. However, Heuper (1959) has induced tumours in mice with dextrans of a higher molecular weight, while Powell and Turner (1961) have reported connective tissue hypertrophy at the injection site of low molecular weight dextran and some tumours have developed in these animals (unpublished observations). Furthermore, Powell (1961) has demonstrated that under certain conditions dextran has severe toxic effects on HeLa cells *in vitro*, and subsequent experiments have confirmed his suggestion that this may be due to the chelating properties of dextrans (Powell and Milner, 1961). Tumours have also been induced *in vivo* by other soluble polymeric substances generally considered to be relatively inactive biochemically (Heuper, 1957 ; Boyland, Charles and Gowing, 1961 ; Lusky and Nelson, 1957).

Experiments by Lundin (1961) have emphasized the importance of the molecular size of iron-organic complexes in carcinogenesis, while Boyland (1960) has suggested that the molecular size may be further increased by iron cross-linking between the dextran molecules. However it seems likely, as Richmond (1961) has suggested, that the dextran functions differently, facilitating the uptake by the cell of an abnormally high concentration of iron. It is clear from Powell's observations (1961) that dextran readily permeates cells *in vitro*, while unpublished experiments by Powell and Turner have shown iron-dextran to be better tolerated both *in vivo* and *in vitro* than other iron compounds at lower iron concentrations. Lundin (1961) has envisaged the possibility that the macromolecular component may ensure a slow release of intracellular iron, but Muir and Goldberg (1961) have studied the uptake of iron-dextran by macrophages *in vivo*, and have found that the complex is taken up as a whole into vacuoles and the dextran eliminated, leaving the iron stored as ferritin granules. This may be true only of macrophage-like cells. In the present observations the amoeboid cells of the $\beta$ response showed iron stored in discrete particles, while in the epithelialised $\alpha$ cells the iron was finely and evenly dispersed. In both cases subsequent cell division rapidly depleted viable cells of iron deposits, and the cells returned to a normal morphology.

### SUMMARY

Fowl fibrocytes, subjected to sub-lethal iron-dextran treatment, have been found to react morphologically in two distinct ways. The response in young cultures is epithelial in nature, resembling accounts of known malignant change in fibrocytes. In older cultures a less specific amoeboid response occurs. The question of physiological stability in cell strains has been raised, and consideration given to the action of dextran in carcinogenesis.

The author wishes to acknowledge the technical assistance of Mr. G. A. Butcher and Miss A. Ross. The expenses of this work were defrayed from a block grant by the British Empire Cancer Campaign.

### REFERENCES

Boyland, E.—(1960) *Progr. exp. Tumour Res.* (Basle, Karger), **1**, 163.
*Idem*, Charles, R. T. and Gowing, N. F. C.—(1961) *Brit. J. Cancer*, **15**, 252.
Earle, W. R.—(1943) *J. nat. Cancer Inst.*, **4**, 165.
*Idem* and Voegtlin, C.—(1940) *Publ. Hlth Rep., Wash.*, **55**, 303.
Ebner, K. E., Hageman, E. C. and Larson, B. L.—(1961) *Exp. Cell Res.*, **25**, 555.
Goldblatt, H. and Cameron, G.—(1953) *J. exp. Med.*, **97**, 525.
Haddow, A. and Horning, E. S.—(1960) *J. nat. Cancer Inst.*, **24**, 109.
Hampton, E. G. and Eidinoff, M. L.—(1962) *Cancer Res.*, **22**, 1061.
Hayflick, L. and Moorhead, P. S.—(1961) *Exp. Cell. Res.*, **25**, 585.
Heuper, W. C.—(1957) *Cancer*, **10**, 8.—(1959) *Arch. Path.*, **67**, 589.

LASNITSKI, I.—(1958) In ‘ Cancer ’, London (Butterworth & Co.), Vol. **3**, p. 42.
LUNDIN, P. M.—(1961) *Brit. J. Cancer*, **15**, 838.
LUSKY, M. L. AND NELSON, A. A.—(1957) *Fed. Proc.*, **16**, 318.
MUIR, A. R. AND GOLDBERG, L.—(1961) *Quart. J. exp. Physiol.*, **46**, 289.
PAUL, J.—(1962) *Cancer Res.*, **22**, 431.
POWELL, A. K.—(1961) *Brit. J. Cancer*, **15**, 354.
*Idem* AND MILNER, A.—(1961) *Rep. Brit. Emp. Cancer Campgn*, **39**, 209.
*Idem* AND TURNER, C. J.—(1961) *Ibid.*, **39**, 206.
RICHMOND, H. G.—(1957) *Scot. med. J.*, **2**, 169—(1959) *Brit. med. J.*, i, 947.—(1960) In ‘ Cancer Progress ’, London (Butterworth & Co.), p. 24.—(1961) *Brit. J. Cancer*, **15**, 594.
SANFORD, K. K., LIKELY, G. D. AND EARLE, W. R.—(1954) *J. nat. Cancer Inst.*, **15**, 215.
SAXÉN, E. AND PENTINNEN, K.—(1958) *Ann. Med. exp. Fenn.*, **36**, 27.
SCOTT, D. B. M., PAKOSKEY, A. M. AND SANFORD, K. K.—(1960) *J. nat. Cancer Inst.*, **25**, 1365.
WEISS, P. (1953) *J. Embryol. exp. Morph.*, **1**, 181
WILLMER, E. N.—(1945) In ‘ Essays on Growth and Form ’, Oxford (Clarendon Press), p. 264.